# Short-Time Germination Modifies Chickpea Grains and Enhances the Production of High-Quality Functional Beverages: Insights into the Soaking Stage in Transformation Processes

**DOI:** 10.1021/acsomega.5c00900

**Published:** 2025-05-06

**Authors:** Juliana Alves Diniz, Sara Aparecida Mendes Diniz Antonio, Ana Carolina Batista da Silva Lemos, Lara Campos Borim, Matheus Monaco, Sinézio Inácio da Silva Júnior, Olga Luisa Tavano

**Affiliations:** † ProtHea – Research Group on Proteins for Health Promotion, 74347 Federal University of Alfenas, 37130-001 Alfenas, Minas Gerais, Brazil; ‡ Graduate Program in Nutrition and Longevity, PPGNL, Federal University of Alfenas, 37130-001 Alfenas, Minas Gerais, Brazil; § School of Nutrition, Federal University of Alfenas, 700 Gabriel Monteiro da Silva Street, 37130-001 Alfenas, Minas Gerais, Brazil; ∥ Food and Medicines Department, School of Pharmaceutical Sciences, Federal University of Alfenas, Federal University of Alfenas, 700 Gabriel Monteiro da Silva Street, 37130-001 Alfenas, Minas Gerais, Brazil

## Abstract

The germination process of grains is a simple and cost-effective method to enhance the food matrix, facilitating the release of additional or novel bioactive components that can be incorporated into products derived from these grains. However, prolonged processes, while effective, are labor-intensive and may increase the risk of product contamination. In this study, we investigated the transformations in chickpeas (var. BRS Cristalino) following 12 h of soaking and 1 day of germination, as well as their effects on the production of aqueous extracts, aiming to improve chickpea-based beverage formulations. The results revealed that the soaking step alone led to significant changes in the soluble components. There was a notable increase in amylase and protease activities, with the latter increasing 5-fold. The total soluble glucose in the extract increased by 20% after soaking, and the protein digestibility improved by 30%. Germination further enhanced digestibility, reaching approximately 50% more than that observed in the untreated grains. Similarly, both soaking and germination altered the trypsin inhibitory activity of the extracts, reducing it by 45 and 80%, respectively, compared with the extract produced from raw chickpeas. Additionally, the antioxidant activity of the extracts increased with both soaking and germination, reaching a 200% increase when evaluated using the DPPH assay. Although no enhancement in ACE and α-glucosidase inhibitory activities was observed, all extracts demonstrated an inhibitory potential. The data suggest that soaking, a common step preceding germination, already induces initial modifications in the grains, altering the solubility of their components and resulting in distinct aqueous extracts. These findings indicate that germination could be a promising process for producing beverages with functional properties. They also highlight the need for further investigation into the effects of even shorter soaking and germination times.

## Graphical abstract

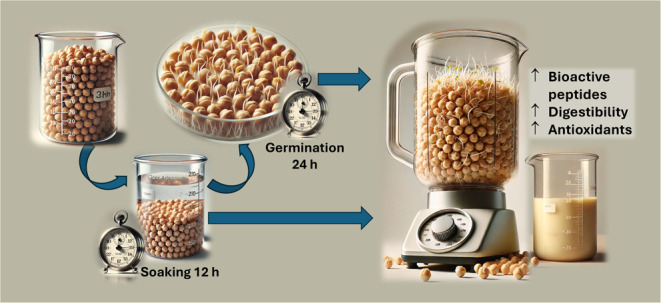

## 1 Introduction

As highlighted by Grossmann and McClements,^1^ solubility is a critical property of legume components, particularly regarding their application in novel food formulations such as plant-based beverages. Among the constituents of legumes, a significant proportion is water-soluble, making the production of aqueous extracts a fractionation process that isolates specific compounds. Consequently, the solubility profile of legume components directly influences the composition of the resulting food product. The solubility profile can vary depending on the developmental stage of the grains.

For instance, one key component that undergoes changes during the formation, development, and subsequent germination of legume grains is protein. Protein solubility in water depends on its structural characteristics, and based on this property, proteins have been traditionally classified^2^ into albumins (soluble in water), globulins (soluble in dilute salt solutions), prolamins (soluble in ethanol solutions), and glutelins (soluble in dilute alkali). Albumins typically account for approximately 20% of the total protein content in legumes.^2^ Additionally, legume proteins can be categorized into storage proteins, biologically active proteins, and structural proteins, all of which play distinct roles in grains and undergo changes during germination and plant development.^2^

These changes reflect both the release of certain proteins and the production of new ones, including the generation of free amino acids and peptides derived from pre-existing proteins mobilized by seed enzymes. Such modifications alter the overall solubility of the protein matrix and may enable novel applications. Increased protein solubility can yield protein-enriched beverages by incorporating previously insoluble protein fractions into the total protein content of the extract. Beyond classical nutritional benefits, novel peptides may emerge with bioactive properties, potentially promoting human health.^3^ The proteases present in grains can differ from those used during digestive proteolysis or external enzymatic hydrolysis, producing unique peptides. Autohydrolysis occurring during seed germination offers a promising approach for releasing soluble and bioactive components.^4^

Germination is thus regarded as a simple and cost-effective process capable of inducing significant changes in legumes and cereals, often enhancing their nutritional and functional properties.^4,5^ In the context of an aging and growing global population, these enhancements are particularly relevant. Older adults often face dietary challenges, including increased protein requirements, the need for easier-to-digest foods, and the demand for foods capable of managing or mitigating age-related conditions such as chronic non-communicable diseases.^3^ Furthermore, difficulties in food intake make beverages an ideal alternative.

Legumes offer the potential for beverages that go beyond protein delivery, as other beneficial compounds such as minerals, oligosaccharides, and flavonoids can also be solubilized and made bioavailable.^6^ As previously mentioned, germination is advantageous because it can be performed at home. However, despite its simplicity, proper hygiene measures are necessary to ensure the safety of the final product. Shorter germination times could be more practical, cost-effective, and conducive to maintaining sanitary conditions. Studies indicate that significant improvements in legume properties can occur within short germination periods. Villeneuve et al.^7^ observed a clear alteration in the nutritional composition and bioactivities of whole flax grains germinated for just 1 day, with free amino acid content and antioxidant activity nearly doubling. Similarly, Bueno et al.,^8^ when comparing different germination times in soybeans, found that just 24 h of germination following an 8 h soaking period was sufficient to induce significant changes in protein, amino acid, and sugar levels. The same authors also noted that many modifications had already occurred during the soaking stage. They observed a 30% increase in water-soluble phenolics and flavonoids after soaking, with these levels remaining unchanged during subsequent germination periods (up to 180 h). This finding suggests that both soaking and short germination times can significantly impact the composition of legumes.

In this study, the effects of short germination times on chickpeas were evaluated, along with the soaking stage preceding germination, as a potential strategy to enhance their functional properties and facilitate the development of simple, accessible aqueous extracts for plant-based beverages.

## 2 Materials and Methods

### 2.1 Germination Process

Chickpea germination was prepared as reported by Bueno et al.^8^ with modifications. Briefly, grains were washed with distilled water, immersed for 1 min in sodium hypochlorite solution at 0.07 g/100 mL and rinsed twice with sterile distilled water. Then, the grains were soaked in distilled water for 12 h at room temperature. After soaking, the grains were germinated on sieves at room temperature for 24 h. Distilled water was sprinkled on the grains after 12 h. Fresh chickpea grains were immediately collected after the soaking stage and after 24 h of germination and used for extractions (as described in Section 2.3).

### 2.2 Moisture and Total Solids

Moisture content was estimated gravimetrically by drying at 105 °C until a constant weight was achieved. The remaining residue was considered to be total solids, while the weight loss was attributed to moisture.

### 2.3 Extracts Preparations

Aqueous extracts were prepared by grinding 1 g of wet in nature (no soaked nor germinated), soaked, and germinated grains with 20 mL of distilled water for 20 s, using Ultraturrax (HomoMix D-500, Biosystems, Curitiba, Paraná, Brazil). The samples were centrifuged at 7000*g* for 20 min/5 °C using a refrigerated centrifuge (FANEN Excelsa 4-MOD 280R-São Paulo, Brazil). The supernatants were used for determinations.

### 2.4 Protein Determination

Nitrogen was determined according to the Kjeldahl method^9^ and protein was calculated as nitrogen × 6.25.

### 2.5 α-Amino Groups Determination

α-Amino groups were determined spectrophotometrically (UV–vis spectrophotometer BEL photonic UV-M51, made in PRC), using OPA (o-phthaldialdehyde) reagent, as described by Church et al.^10^ Briefly, aliquots of the samples between 0 and 130 uL were added to 1 mL of OPA reagent, prepared every day (25 mL of 100 nmol/L sodium tetraborate, 2.5 mL of SDS 20%, 40 mg of OPA in 1 mL of methanol, 100 uL of α-mercaptoethanol, and adjusted to 50 mL with distilled water). After exactly 2 min of reaction, the absorbance was measured at 340 nm using the reagent solution as a blank. Analytical reference curves were constructed by using l-leucine as the standard.

### 2.6 SDS-PAGE

SDS-PAGE was carried out as described by Laemmli,^11^ using a 12.0 g/100 mL polyacrylamide gel as separating gel and a 4.0 g/100 mL stacking gel. The samples were premixed, at a 1:1 ratio, with sample buffer containing 0.5 mol/L Tris–HCl buffer pH 6.8, 1 g/100 mL bromophenol blue, 10 g/100 mL glycerol, and 2 g/100 mL SDS. The gels were stained with Coomassie brilliant blue G-250 and destained using a methanol-acetic acid solution.

### 2.7 Amylase and Protease Activity

Amylase activity in the aqueous extracts was determined using starch as a substrate (1 g/100 mL in 100 mmol/L of citrate-phosphate buffer at pH 6.0). Starch hydrolysis was monitored at 37 °C by measuring reducing sugar using dinitrosalicylic acid method at 540 nm, as described by Miller.^12^ Protease activities were verified by the proteolytic activity performed using benzoyl-dl-arginine-*p*-nitroanilide (BAPNA) as substrate, as described by Kakade et al.^13^

### 2.8 *In Vitro* Protein Digestibility

*In vitro* protein digestibility of 50 mg of protein per sample was determined as described by Akeson and Stahman^14^ using a pepsin-pancreatin incubation sequence (0.75 mg pepsin and 2.0 mg pancreatin), at 37 °C, for 3 and 24 h, respectively. The enzymatic reaction was then interrupted by adding trichloroacetic acid (TCA) until a final concentration of 10 g/100 mL was reached, followed by centrifugation at 7000 *g* for 15 min. The supernatant was used in the determination of free α-amino groups, determined using the OPA reagent, as previously described in Section 2.5. Results were expressed as a perceptual of disrupted peptide bonds, considering that each α-amino group released represents one hydrolyzed bond. The 100% hydrolysis degree was estimated by the total protein mass in the sample and the average molecular weight of amino acids (MW = 113), whereas each amino acid contains an α-amino group, and each one represents a potential bond to be disrupted.

### 2.9 Trypsin Inhibitors

Trypsin inhibitors were measured as described by Kakade et al.^13^ Briefly, 2 mL of the extract or 2 mL of distilled water (for 100% trypsin activity) were mixed with 2 mL of a standard bovine trypsin (Sigma Type I) solution and incubated for 10 min at 37 °C. Then, 5 mL of prewarmed benzyl-dl-arginine-*para*-nitroanilide (BAPNA) was added and incubated for another 10 min at 37 °C. The reaction was stopped by adding 30% acetic acid. Absorbance was measured at 410 nm. One trypsin inhibitor unit (TIU) was arbitrarily defined as a 0.01 reduction in absorbance. Results are expressed as trypsin inhibitor units (TIU) per gram of protein or sample.

### 2.10 Free Glucose Concentration

Free glucose was determined using a colorimetric peroxidase-glucose oxidase kit (Glucose Monoreagent-BioClin-K082). Glucose curve references were used (0.5–5.0 μg/mL).

### 2.11 Total Phenolic and Flavonoid Content

Total phenolic was determined spectrophotometrically (UV–vis spectrophotometer BEL photonic UV-M51, made in PRC), using the Folin-Ciocalteu reagent, according to the Boateng et al.^15^ A standard curve of gallic acid was constructed (0–16 μg/mL), and the results were expressed as gallic acid equivalent (GAE) per gram of samples. Flavonoid contents were determined using aluminum chloride spectrophotometric assay as described by Boateng et al.^15^ A catechin curve was used as standard (0–50 μg/mL), and the results were expressed as milligrams of catechin equivalent (CE) per gram of samples.

### 2.12 Potential Antioxidant Activity

ABTS^+^ radical was generated by incubation of 7 mmol/L ABTS with 2.4 mmol/L potassium persulfate for 16 h in the dark, as described by Shalaby and Shanab.^16^ The ABTS cation solution was diluted with water until an absorbance of 0.700 in spectrophotometer, at λ = 734 nm. To 250 μL of sample was added 750 μL of an ABTS solution. Absorbance was read after 60 min in the dark. DPPH Radical-Scavenging activity according to Brand-Williams et al.^17^ with some modifications. Briefly, 0.1 mL of sample extract was added to 3.9 mL of an 80/100 mL ethanol DPPH solution. The absorbance was determined at 517 nm. A standard curve was prepared using TROLOX as reference, and results were expressed as μmol of TROLOX equivalent/g of sample for both assays (1.0–7.0 nmol/mL).

### 2.13 Angiotensin-Converting Enzyme Inhibition

Angiotensin-converting enzyme (ACE) inhibitory activity was performed based on the method described by Holmquist et al.,^18^ using FAPGG (N-[3-(2-furyl) acryloyl]-l-phenylalanyl-glycyl-glycine) as a substrate, with some modifications: 10 μL of ACE -F7131-Sigma (50 mU·mL^–1^) and 40 μL of extracts. After 5 min, 750 μL of FAPGG (0.5 mM) was added, and the reaction was monitored at 340 nm for 30 min, with readings taken at zero time and every 5 min. To the noninhibited ACE activity (100% of activity) distilled water was used replacing the extract. The results were expressed as % inhibitory activity.

### 2.14 α-Glucosidase Inhibition

α-Glucosidase inhibitory activity was performed based on the method described by Toma et al.,^19^ with some modifications: 10 μL of α-glucosidase and 10 μL of extracts were incubated for 10 min and then 20 μL of starch 1% was added. After 20 min/37 °C, hydrolysis was monitored by determination of free reducing sugar using dinitrosalicylic acid method at 540 nm, as described by Miller.^12^ To the noninhibited enzyme activity (100% of activity), distilled water was used replacing the extract. The results were expressed as % inhibitory activity.

### 2.15 Statistical Analyses

Data were analyzed with JASP software (version 0.19.1, 2023). The Shapiro–Wilk test was employed to assess normality, Pearson’s test was used to examine correlations, and ANOVA with Tukey’s post hoc test was conducted for mean comparisons. A significance level of *p* < 0.05 was adopted.

## 3 Results and Discussion

Based on the data presented in Figure 1, it can be observed that chickpea grains readily absorb hydration water. Considering that the maximum weight gain after 12 h was, on average, 104.47%, approximately 40% of this water gain was achieved within the first 30 min. It is also noteworthy that within just 4 h, the maximum absorption was nearly complete, with a 96.76% weight gain. This finding is particularly noteworthy, as some authors propose that the initial changes in the grain germination process begin once the initial water absorption phase is completed.^20^

**Figure 1:**
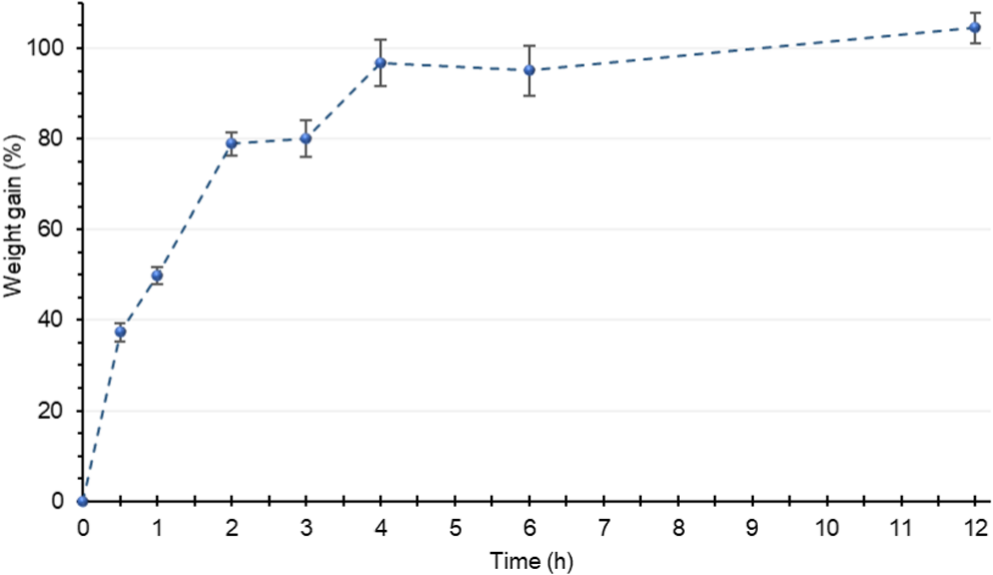
Weight gain of chickpea grains (var. BRS Cristalino) during the soaking process. Results are expressed as the mean and standard deviation (error bars) of three determinations.

There is no consensus in the literature regarding the germination time or the soaking duration in the initial stages. However, many studies adopt an initial hydration period of 12 h, after which different germination methods are applied to hydrated grains. In many cases, the germination time is counted from the state of fully hydrated grains, ignoring changes that may occur during the soaking phase itself. In this study, the grains were analyzed during the early stages of transformation, with initial analyses conducted immediately after the grains were hydrated (12 h).

Further studies are needed to investigate these early stages in greater detail. However, some of our data comparing native grains (time 0, without any processing) to those hydrated for 12 h already suggest that this period was metabolically active, even though no visible changes were observed. Figure 2 illustrates the appearance of the grains before and after 12 h of hydration (Figure 2A,B) and after an additional 24 h of germination, at which point small radicles become visible (Figure 2C,D).

**Figure 2:**
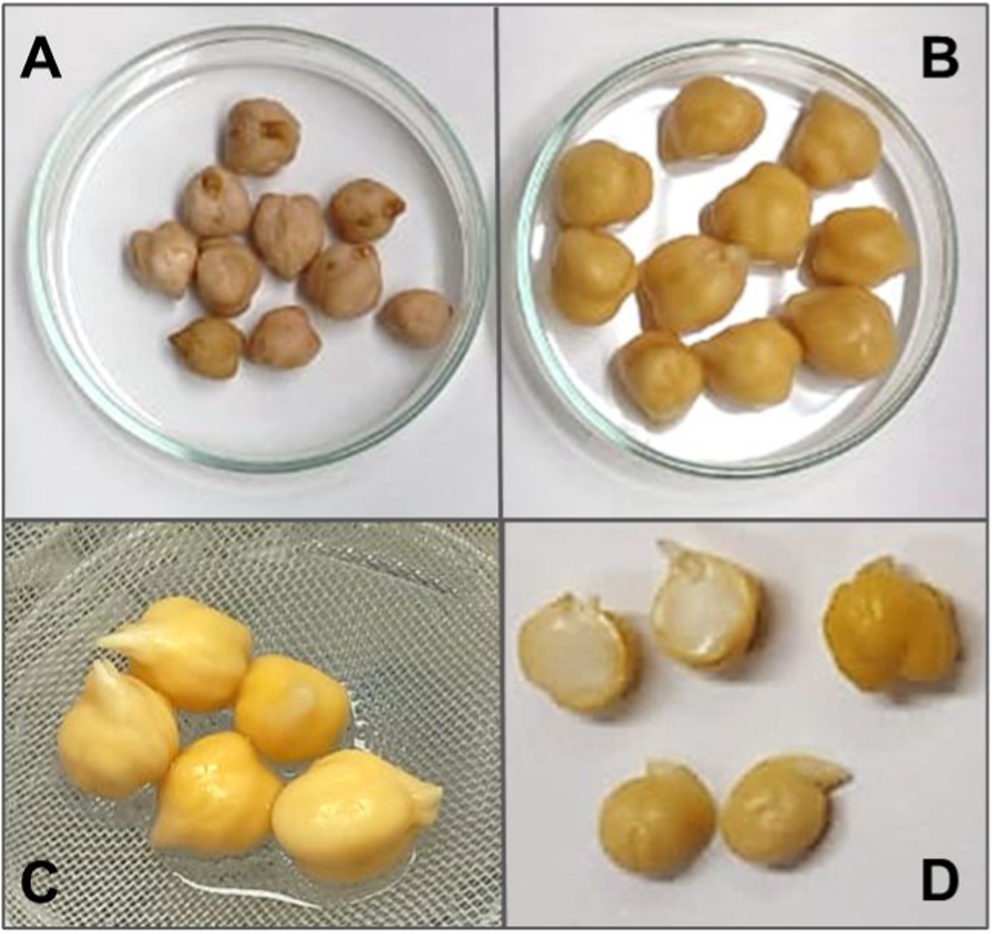
Chickpea grains are BRS Cristalino. (A) *In nature* grains; (B) Grains after 12 h of hydration; (C) Grains after 12 h of hydration followed by 24 h of germination; (D) Grains like in (C) but without coats.

One of the most indicative observations of the significant metabolic activity in the grains is the substantial alteration in the enzymatic profiles of amylases and proteases detected (Figure 3A,B, respectively). After 12 h of hydration, the grains exhibited approximately twice the amylolytic activity, which was maintained after 24 h of germination (Figure 3A).

**Figure 3:**
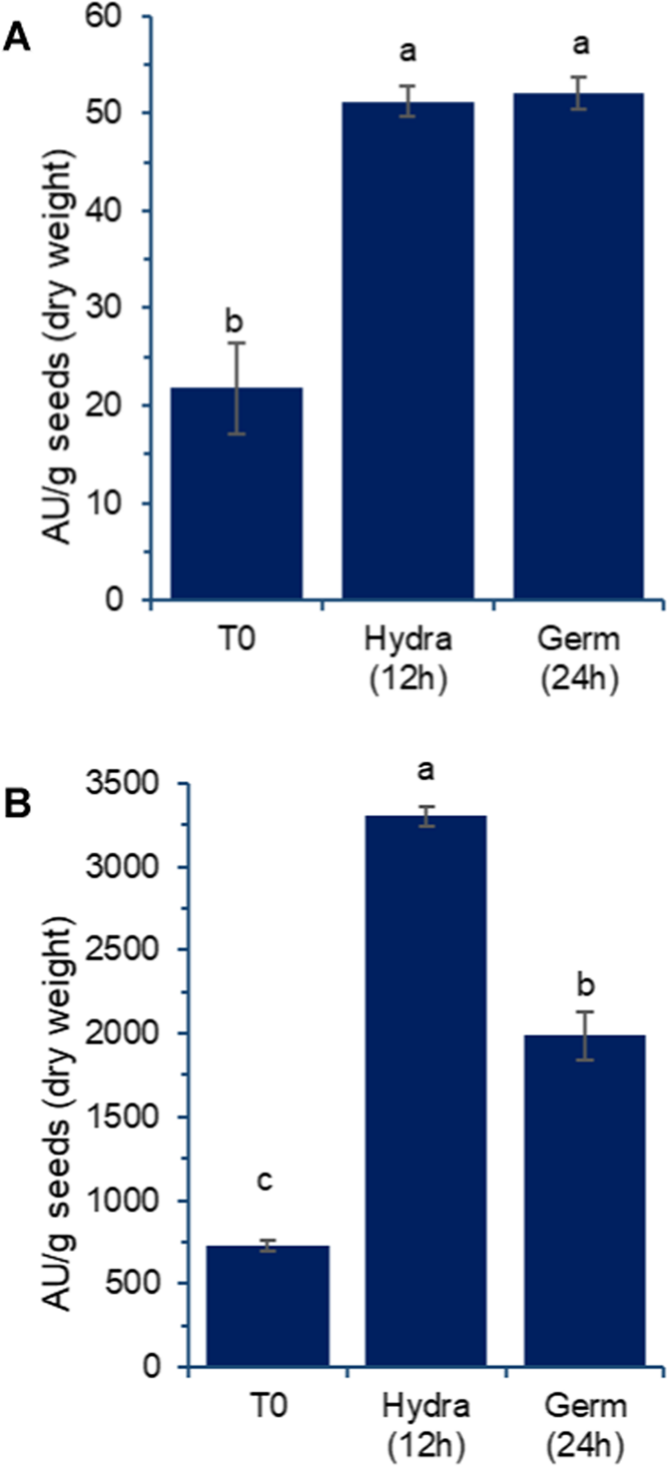
Effect of germination on amylolytic (A) and proteolytic (B) activities detected in aqueous extracts prepared from chickpea grains, var. BRS Cristalino. T0: untreated grains; Hydra (12 h): grains after 12 h of hydration; Germ (24 h): grains after 12 h of hydration followed by 24 h of germination, as described in Section 2. Results are expressed as mean and standard deviation (error bars) of three determinations. Different letters above the bars indicate statistically significant differences between the samples (*p* < 0.05).

Proteolytic activity also increased significantly after soaking, reaching four times the level observed in nonhydrated grains. This activity changed again after 24 h of germination, decreasing significantly but remaining more than twice that of nonhydrated grains (Figure 3B). Safonova et al.,^21^ reported similar increases in amylase and protease activities, with protease activity showing a rapid increase followed by a reduction after 24 h of germination. They also observed a rapid increase in lipoxygenase activity during the initial hours, accompanied by a pronounced decrease in urease activity.

Regarding enzymatic changes during the early germination stages, other studies have also reported significant modifications. Bueno et al.^8^ noted increased protease and amylase activities in germinated soybean grains, particularly during the first hours, stabilizing after the second day. This intense enzymatic activity seems to be consistent across the various grain types. Nam and Park^22^ observed increased proteolytic and amylolytic activities in quinoa, amaranth, and brown rice grains within the first 6 to 12 h of germination.

Future studies should evaluate grains at even earlier time points, as the grains achieved nearly maximum water absorption after approximately 4 h of hydration (Figure 1). This could indicate that significant transformations begin at this stage.

These pronounced changes in enzymatic activities may correlate with alterations in glucose and soluble protein profiles, as observed in the extracts produced (Figure 4). The free glucose content in the grains also increased during germination, showing a significant difference from time zero after 12 h of soaking (Figure 4A).

**Figure 4:**
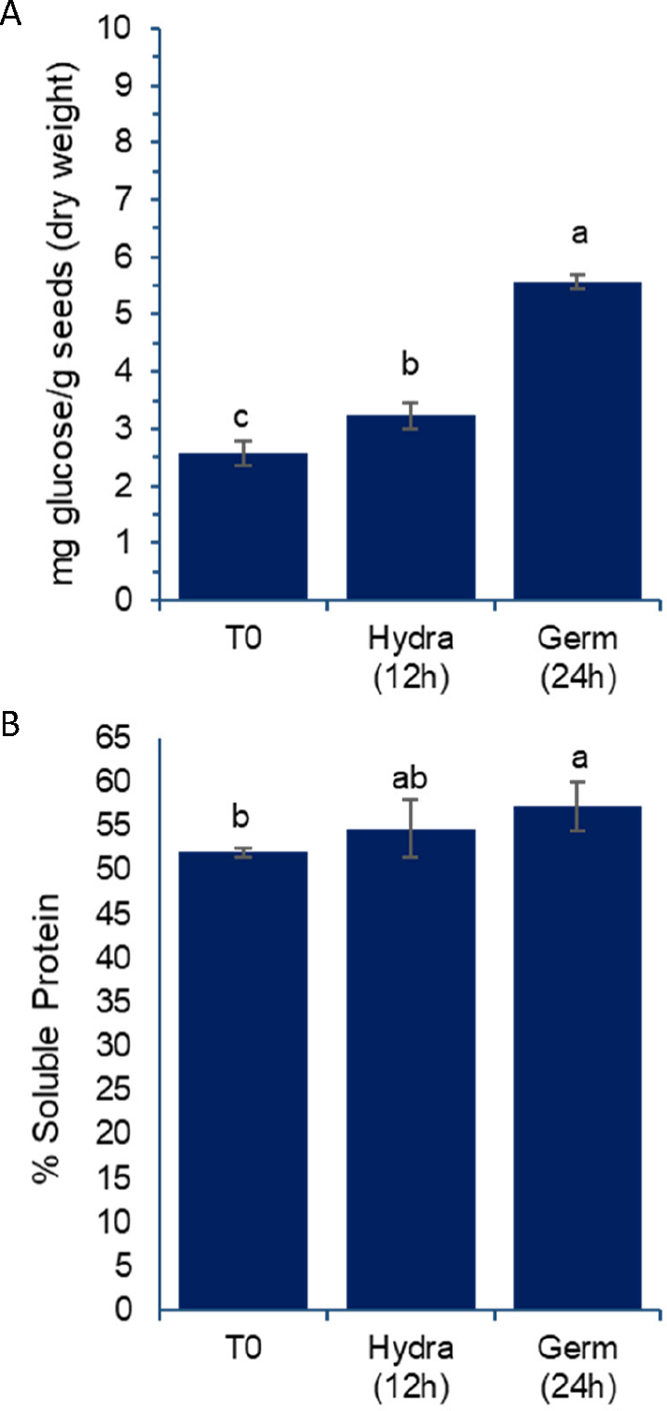
Effect of germination on the percentage of free glucose (A) and soluble proteins (B) in aqueous extracts prepared from chickpea grains, var. BRS Cristalino. T0: untreated grains; Hydra (12 h): grains after 12 h of hydration; Germ (24 h): grains after 12 h of hydration followed by 24 h of germination, as described in the Section 2. Results are expressed as mean and standard deviation (error bars) of three determinations. Different letters above the bars indicate statistically significant differences between the samples (*p* < 0.05).

Although the percentage of soluble proteins did not increase markedly during these short germination periods (Figure 4B), a closer examination of protein profiles suggests intense metabolic activity during the initial 12 h. Figure 5 presents the electrophoretic profiles of the three extracts.

**Figure 5:**
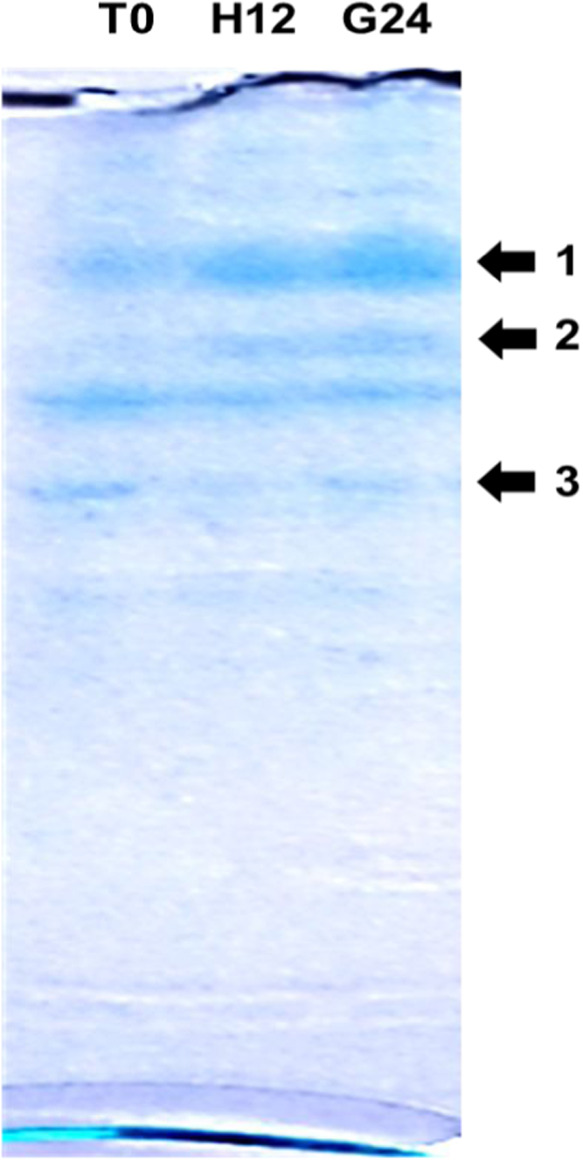
Protein profiles on an SDS-PAGE gel of aqueous extract of different chickpea samples chickpea grains, var. BRS Cristalino. T0: untreated grains; H12: grains after 12 h of hydration; G24: grains after 12 h of hydration followed by 24 h of germination, as described in the Section 2.

While the overall electrophoretic profiles are similar, certain bands indicate changes in the types of soluble proteins released by the grains. For example, the bands marked by arrows 1 and, particularly, 2 appear more intense after 12 h of hydration. In contrast, band 3 decreases in intensity and then increases again over time. These observations may be related to the emergence or release of new enzymatic chains.

This accelerated metabolic activity in the grains may also be related to the protein digestibility results. Table 1 shows the percentage of hydrolysis by digestive enzymes (pepsin-pancreatin) compared with casein as a standard.

**Table 1:** Effect of Germination on *In Vitro* Digestibility of Proteins from Aqueous Extracts Prepared from Chickpea Grains, var. BRS Cristalino^a^

sample	hydrolysis degree (%)	% of casein result
T0	13.34 ± 1.56^d^	50.89
hydra 12 h	17.10 ± 0.68^c^	65.21
germ 24 h	19.90 ± 0.22^b^	75.90
casein	26.22 ± 1.30^a^	100

a T0: untreated grains; Hydra (12 h): grains after 12 h of hydration; Germ (24 h): grains after 12 h of hydration followed by 24 h of germination, as described in the Section 2. Values are means ± SD, *n* = 3. Different letters among the degrees of hydrolysis indicate statistically significant differences (*p* < 0.05).

Table 1 data indicate a significant improvement in digestibility during the soaking phase, which continues to increase, reaching a 50% improvement compared to T0 (untreated grains). Sofi et al.^5^ also reported a notable increase in protein and starch digestibility in two desi chickpea varieties after 12 h of soaking and 36 h of germination. Uppal and Bains^23^ observed similar improvements after soaking for 12 h and germinating for 36 h, and other studies have demonstrated increased digestibility with longer germination periods, such as 72 h.^24^

*In vitro* digestibility assays essentially reflect the ease with which sample proteins are hydrolyzed upon contact with digestive enzymes. This hydrolysis potential can be influenced by various factors, including the protein chains themselves or the food matrix with its additional components and physical structure. In this study, as aqueous extracts were analyzed, the influence of the grain matrix was considered minimal. However, the susceptibility of the extracted proteins to hydrolysis and the solubilization of enzyme inhibitors may contribute significantly to these profiles. The SDS-PAGE image in Figure 5 indicates molecular profile changes, likely leading to generally more susceptible forms, consistent with the data in Table 1. Furthermore, the increased presence of free amino acids or small peptides (Figure 6, gray bars) observed during prehydrolysis suggests higher total hydrolysis, indicating more digestible material. Bueno et al.^8^ support this observation. In addition to the enhanced peptide bond breakdown, the samples may contain a greater proportion of low-molecular-weight protein materials. Bera et al.^4^ confirmed significant changes in the molecular weight profiles of various polypeptides and free amino acids after germination of five legume species.

**Figure 6:**
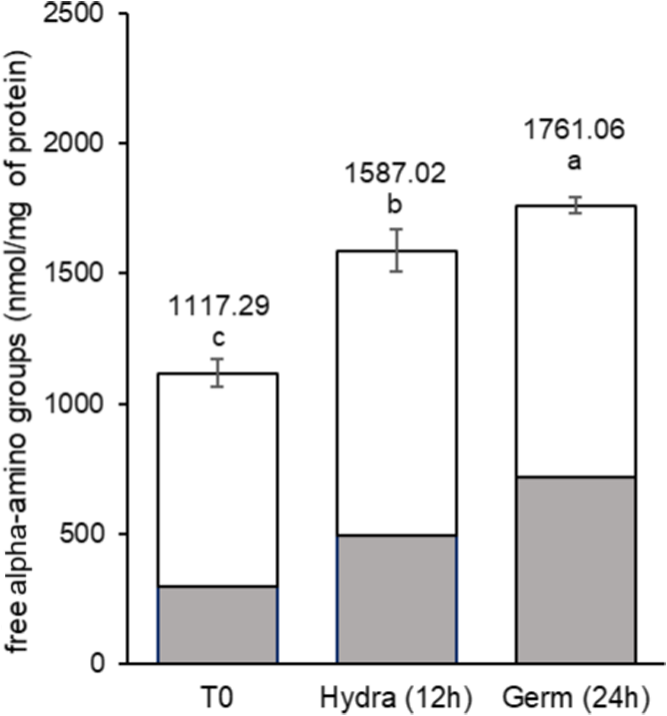
Effect of germination on the degree of protein hydrolysis in aqueous extracts from chickpea grains, var. BRS Cristalino, expressed as free α-amino groups detected before (gray bar) and after (white bar) pepsin-pancreatin digestion. T0: untreated grains; Hydra (12 h): grains after 12 h of hydration; Germ (24 h): grains after 12 h of hydration followed by 24 h of germination, as described in the Section 2. Results are expressed as mean and standard deviation (error bars) of three determinations. Different letters above the bars indicate statistically significant differences between the samples (*p* < 0.05).

At least numerically (nmol/mg of soluble proteins) (Figure 6), this observation holds significance and may lead to additional positive outcomes. Beyond improved digestibility, self-generated peptides can provide distinct advantages related to the bioactivities of the material. The unique protein profile may contribute positively. Peptides are increasingly recognized as bioactive food components capable of supporting various health-promoting effects.^25^

The observed improvements in protein digestibility were expected and may be linked to a reduction in protease inhibitors. Data presented in Figure 7 clearly demonstrates a significant reduction in trypsin inhibitory activity, which begins prominently after soaking and decreases further after an additional 24 h of germination. A reduction of approximately 50% was observed with each processing cycle, whether expressed per gram of grain solids or per milligram of protein in the extracts (Figure 7A,B, respectively).

**Figure 7:**
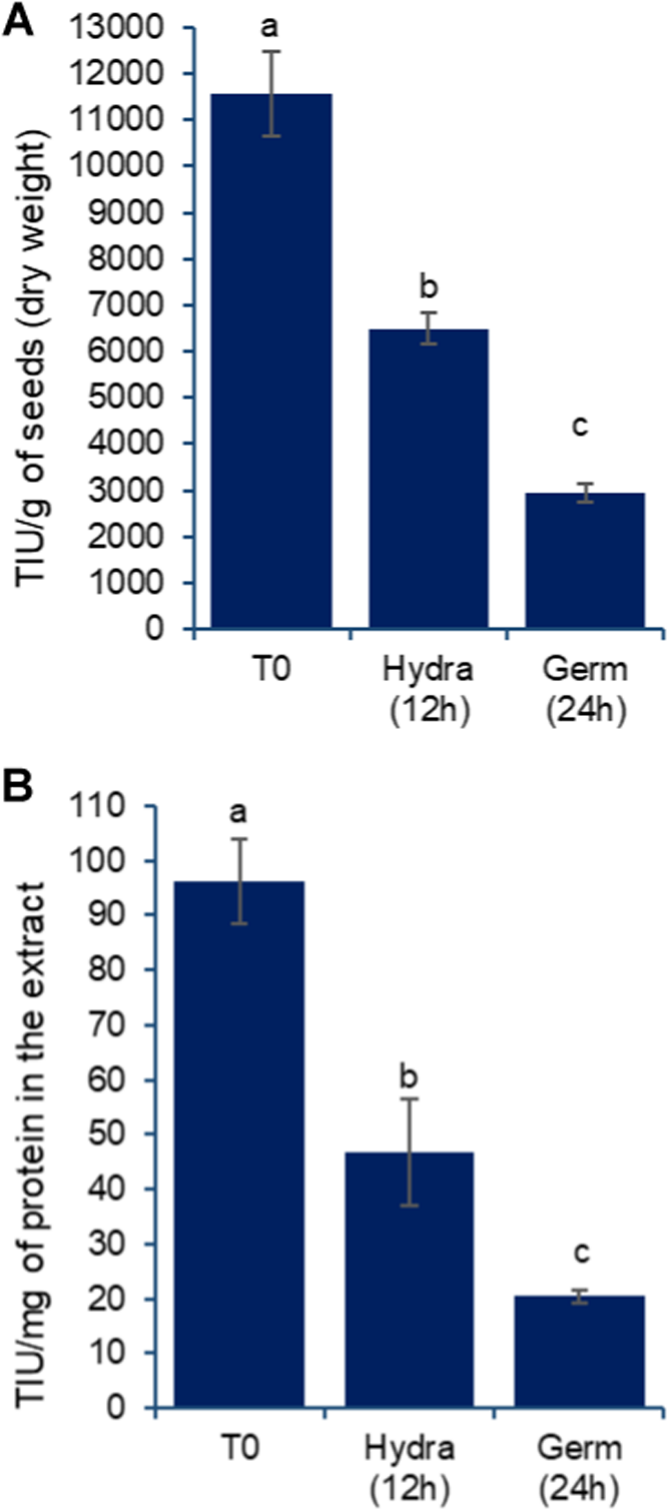
Effect of germination on Trypsin Inhibition Unit (TIU) activity detected in aqueous extracts prepared from chickpea grains, var. BRS Cristalino and expressed per 1 g of dry basis grains (A) and per 1 mg of solubilized protein (B). T0: untreated grains; Hydra (12 h): grains after 12 h of hydration; Germ (24 h): grains after 12 h of hydration followed by 24 h of germination, as described in the Section 2. Results are expressed as mean and standard deviation (error bars) of three determinations. Different letters above the bars indicate statistically significant differences between the samples (*p* < 0.05).

Although the reduction in inhibitory activity is positively associated with improved digestibility, the presence of such inhibitors in food is increasingly viewed as beneficial, as noted in various studies.^26–28^ Consequently, the detection of some remaining inhibitory activity, albeit reduced, may also represent one of the positive bioactivities of these extracts, adding another layer of potential for biofunctional beverage production.

Regarding trypsin inhibition activities detected, it is challenging to determine whether they can be attributed to Bowman-Birk or Kunitz-type inhibitors due to the methodology employed. Previous studies have demonstrated that other grain components, such as phenolic compounds, may also inhibit these enzymes.^29^ However, data from Figure 8, particularly Figure 8A, showing total phenolic content in the extracts indicate a gradual increase in phenolic release. This suggests that the inhibitory activities are less likely to be caused by phenolics and more likely to be attributed to Bowman–Birk inhibitors.

**Figure 8:**
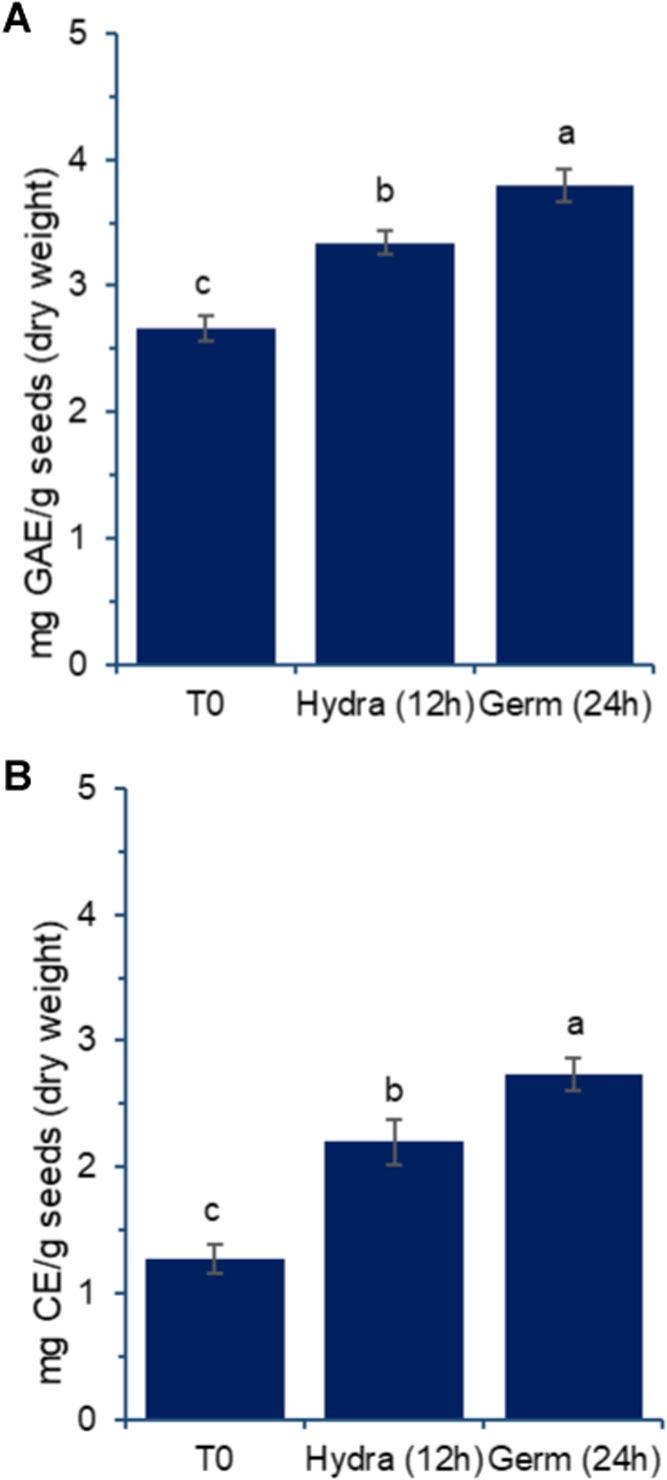
Effect of germination on total phenolic (A) and total flavonoids (B) in aqueous extracts prepared from chickpea grains, var. BRS Cristalino. T0: untreated grains; Hydra (12 h): grains after 12 h of hydration; Germ (24 h): grains after 12 h of hydration followed by 24 h of germination, as described in the Section 2. Results are expressed as mean and standard deviation (error bars) of three determinations. Different letters above the bars indicate statistically significant differences between the samples (*p* < 0.05).

An interesting aspect to discuss is that the soaking step is often considered to enhance the nutritional properties of grains due to the potential removal or reduction of certain compounds through solubilization in hydration water, followed by its disposal. While this process may indeed contribute, it is likely not the sole benefit. Data from Figure 8 does not indicate a reduction; instead, it suggests that while some components may have been solubilized and reduced, others were generated or released. Studies have shown similar increases in the total phenolic content in germinated grains. When individual components are examined; however, they reveal significant changes in the profile rather than just the total content detected.

Sofi et al.^5^ demonstrated that, despite a reduction of over 50% in tannins and phytic acid, the total phenolic content increased after 12 h of soaking followed by 36 h of germination in chickpeas. Additionally, the individual profiles revealed increases in gallic and *p*-coumaric acid levels, while ellagic and cinnamic acids decreased. The same authors highlighted cultivar differences, noting that ferulic acid was undetectable in the GNG 469 cultivar after germination, whereas its levels increased in the GNG 1581 cultivar.

The presence of phenolic compounds in foods has been linked to various health-promoting properties, with many exhibiting diverse bioactivities.^30,31^ Among phenolics, flavonoids are particularly notable and well known for their beneficial effects on human health.^30,32^ Ullah et al.^33^ demonstrated that flavonoids possess antifungal, antidiabetic, neuroprotective, cardioprotective, anticancer, antibacterial, antimalarial, anti-inflammatory, and antioxidant activities. Figure 8B highlights the presence of these components and their progressive increases with each processing step.

These significant enzymatic and molecular changes could also influence the antioxidant activities of the extracts. Figure 9A,B illustrates the antioxidant activities of the extracts, measured using DPPH and ABTS assays, respectively.

**Figure 9:**
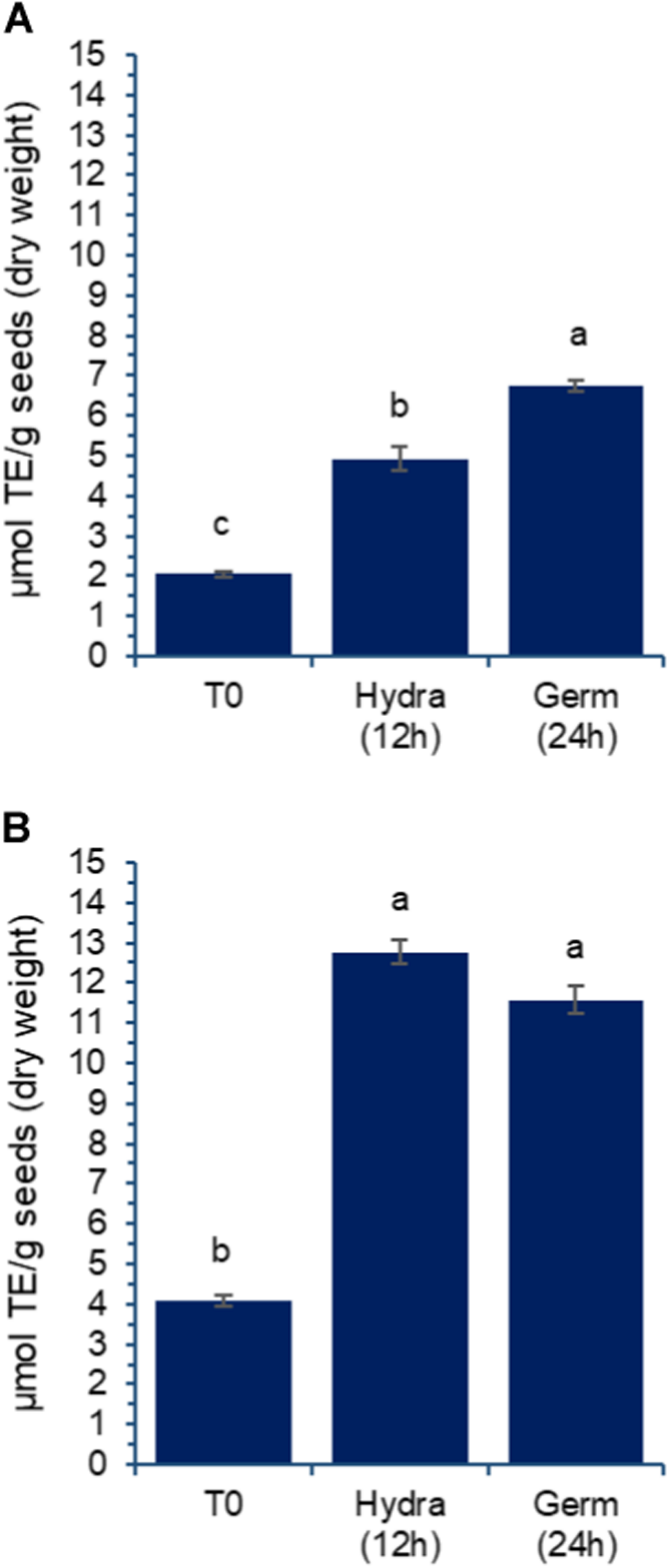
Effect of germination on antioxidant activity detected using DPPH (A) and ABTS (B) in aqueous extracts prepared from chickpea grains, var. BRS Cristalino. TE: TROLOX equivalent; T0: untreated grains; Hydra (12 h): grains after 12 h of hydration; Germ (24 h): grains after 12 h of hydration followed by 24 h of germination, as described in the Section 2. Results are expressed as mean and standard deviation (error bars) of three determinations. Different letters above the bars indicate statistically significant differences between the samples (*p* < 0.05).

The DPPH assay reflects the increasing total phenolic and flavonoid contents shown in Figure 8. Although the ABTS assay data (Figure 9B) indicate no significant changes between hydrated and germinated grains after 24 h, there is a marked improvement in activity following the 12 h soaking period.

It is important to emphasize that the extracts analyzed in this study are aqueous, meaning that the detected components are water-soluble.^34^ Several authors highlight the significance of water solubility for pharmacological and nutraceutical applications of phenolic compounds. Among the wide range of phenolics, solubility and bioactivity variations contribute to different extract applications in human nutrition.^35^ Besides phenolics, other water-soluble components such as vitamins A and E, carotenoids, and peptides may also contribute to the antioxidant activities observed.^36^

In addition to antioxidant activities and the presence of protease inhibitor, other bioactivities may be transferred to the extracts from the grains, whether germinated or not. Figure 10 presents the detected activities for angiotensin-converting enzyme (ACE) inhibition and α-glucosidase inhibition.

**Figure 10:**
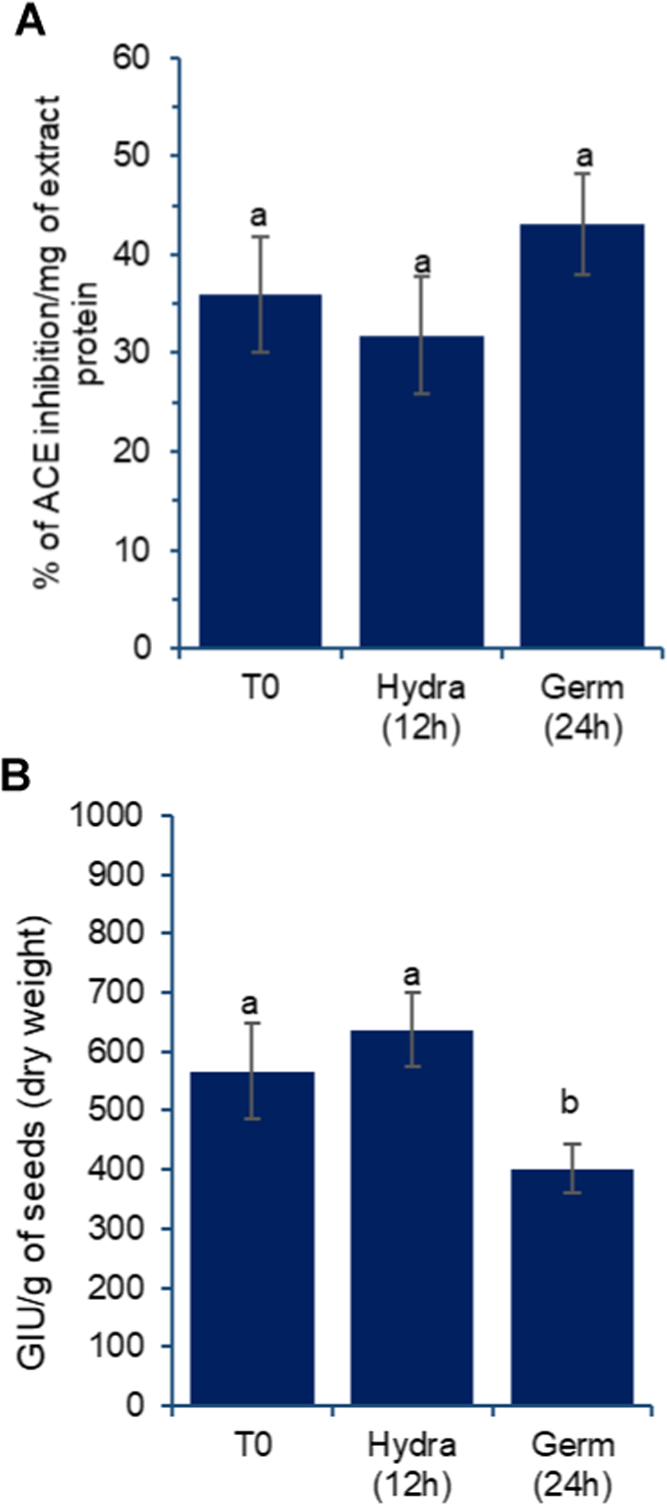
Effect of germination on angiotensin-converting enzyme (ACE) inhibition (A) and α-glucosidase inhibition (B) activities detected in aqueous extracts prepared from chickpea grains, var. BRS Cristalino. T0: untreated grains; Hydra (12 h): grains after 12 h of hydration; Germ (24 h): grains after 12 h of hydration followed by 24 h of germination, as described in the Section 2. Results are expressed as mean and standard deviation (error bars) of three determinations. Different letters above the bars indicate statistically significant differences between the samples (*p* < 0.05).

These bioactivities did not significantly increase, remaining consistent for ACE inhibition (Figure 10A) and slightly decreasing after 24 h of germination for α-glucosidase inhibition. This indicates that the released peptides in the extracts did not achieve sufficient alteration or concentration to impact bioactivities significantly, even though activity was detected in all samples. This differs from findings by other authors, such as Di Stefano et al.,^37^ who observed a marked increase in α-glucosidase inhibition after 3 days of germination.

The results presented here indicate that short germination times may represent promising transformation processes for these raw soluble materials, enhancing their potential to produce functional foods. Even within such short periods, both nutritional and functional improvements were observed in the extracts, suggesting the potential for producing chickpea-based beverages with additional associated benefits. Furthermore, the data highlight that short germination times could be an attractive alternative for both domestic and industrial processes, combining cost reduction with decreased risks of contamination and compromised sanitary quality of the products, as longer processes may require more stringent microbiological control of the grains. Additionally, the interpretation of the benefits associated with the soaking stage was raised, contributing to further discussions on the topic. But studies aimed at understanding the levels of transformation and the minimum time required to observe the positive effects of this process need to be conducted in the future with both longer and shorter time intervals. Additionally, parallel studies on the organoleptic quality of potential beverages should be conducted to assess their consumability.

## Abbreviations

DPPH 2,2: diphenyl-1-picrylhydrazyl
ABTS 2,2′: azino-Bis (3-ethylbenzothiazoline-6-sulfonic acid) diammonium aalt
TROLOX 6: hydroxy-2,5,7,8-tetramethylchroma Ne-2-carboxylic acid
ACE: angiotensin-converting enzyme
